# Diseases That Never Co-exist

**Published:** 1842-12-03

**Authors:** 


*Diseases that never co-exist.*—Some very interesting results have been obtained by Professor Rokitansky, on the incompatibility of certain diseases with each other. From the earliest ages a vague idea has prevailed that two diseases could not co-exist in the system. This opinion was thus far modified by John Hunter, who says that "no two actions from two different morbid poisons can go on together at the same time, in the same part, or the same constitution." Later observations, while they have shown this statement, as expressing a general law, to be erroneous, have at the same time indicated, that certain diseases exert upon others an opposing influence, in the way of the one arresting the course, in modifying the nature, of the other. For example, measles and small-pox have been observed to suspend, or modify, the course of each other. Hooping-cough sometimes suspends the small-pox, measles, and scarlet fever. Hooping-cough is frequently cured by vaccination. It is sometimes also cured by small-pox and measles. Vaccinia may suspend, or in its turn be suspended by, scarlatina. The plague was arrested by the prevalence of small-pox, but broke out again on its disappearance, according to Baron Larrey.

But these and other isolated facts were never of a sufficiently definite character to attract much attention; and it remained for Professor Rokitansky, whose unequalled opportunities of observation, and whose acknowledged accuracy create the most perfect confidence in his investigations, to put this matter wholly in a new light, by establishing, from an amount of cases that renders fallacy in the result almost impossible, that certain diseases never co-exist; as the presence of the one arrests the progress, or prevents the occurrence, of the other. The typhus abdominalis (that is, with formation of the characteristic typhous matter, and which by Rokitansky is always understood under the name of typhus,) is excluded by the various forms of puerperal fever. In two hundred dissections of puerperal fever he did not find one complication of the typhous process. This immunity from typhus is given by the pregnant state, child-bed, and even, though in a less degree, by suckling.

Typhus and cholera, and typhus and dysentery, are said to have the power of mutual exclusion; and the co-existence of tuberculous disease and typhus is extremely rare. Carcinoma and tuberculosis (i. e., tuberculous disease) are antagonist diseases; and the latter, and all kinds of serous cysts, are never met with simultaneously in the same organ, or even in the same individual. Tubercular disease affords an imuunity from cholera, dysentery, hypertrophy of the heart, curvature of the spine, dilated bronchia, and almost all chronic diseases of the stomach. Tuberculosis and aneurism do not co-exist, and Rokitansky, as well as others, has remarked, that the developement of tubercle is arrested, although the disease is not subdued, by the pregnant state, as likewise by all large tumours of the abdomen. These conclusions are derived by Rokitansky from numerous post-mortem and other examinations; and although exceptions may occasionally occur, yet if their main truth be established by subseqnent researches, they may be rendered available in the history and treatment of various affections, and furnish a text for more extended inquiries by future pathologists.—*Med. Chir. Rev.*, Oct. 1842.

